# Performance of DRNNAGE for macroscopic age‐at‐death estimation in contemporary Brazilian identified skeletons

**DOI:** 10.1111/1556-4029.70344

**Published:** 2026-04-24

**Authors:** André Marquim Nogueira da Fonte Cornélio, David Senhora Navega, Marcus Vitor Diniz de Carvalho, Tatyane dos Santos Ferreira, Carolina Peixoto Magalhães, Eugénia Cunha, Evelyne Soriano

**Affiliations:** ^1^ Postgraduate Program in Forensic Sciences University of Pernambuco (UPE) Recife Brazil; ^2^ Panacea Cooperative Research S. Coop Ponferrada (León) Spain; ^3^ Centre for Functional Ecology (CFE), Department of Life Sciences, Laboratory of Forensic Anthropology University of Coimbra Coimbra Portugal; ^4^ Postgraduate Program in Forensic Sciences, Center for Studies in Forensic Anthropology (CEAF) University of Pernambuco (UPE) Recife Brazil; ^5^ Laboratory of Human Identification and Forensic Osteology (LIHOF) Federal University of Pernambuco (UFPE) Vitória de Santo Antão Brazil

**Keywords:** age estimation, DRNNAGE, forensic anthropology, human identification, neural networks

## Abstract

In global forensic literature, methods for estimating age in young individuals are more commonly addressed than those applicable to adults. This trend is also evident in Brazil. This study aimed to evaluate the performance of the DRNNAGE software for age estimation in Brazilian identified human skeletons. The sample consisted of 400 skeletons (200 females and 200 males), aged 20 years and older, from the osteological collection of the Center for Studies in Forensic Anthropology at the University of Pernambuco (CEAF/UPE). The DRNNAGE trait scoring system was applied to analyze morphological features of the cranial sutures, vertebrae, upper and lower limbs, fusion of the first and second sacral vertebrae, clavicles, first rib, pubic symphysis, sacroiliac joint, and acetabulum. Cohen's Kappa was used to assess inter‐ and intra‐observer agreement. Descriptive and statistical analyses were performed in IBM™ SPSS® (version 22.0), R (version 4.3.3), and RStudio IDE (version 2023.12.1). Neither sex nor antimeric variation influenced the classification of bone maturation stages. DRNNAGE achieves 6.54‐year MAE and 90.2% empirical coverage (90% nominal) in a Brazilian sample. The software performed satisfactorily in this sample and reinforces the importance of further regional studies to assess its nationwide applicability, considering Brazil's continental dimensions and diverse population structure, taking into account the well‐known intra‐ and inter‐population variability in human aging patterns.

**Highlights**

- DRNNAGE achieves an MAE of 6.54 years and 90.2% prediction coverage (nominal confidence of 90%).
- Higher skeletal incompleteness in females reduced prediction coverage compared to males.
- The method remained robust even when trait availability dropped to the lower end of 34%.
- Systematic underestimation bias increases in individuals over 80 years of age.
- DRNNAGE is a valid, open‐source tool suitable for casework involving incomplete remains.

## 1 INTRODUCTION

In forensic anthropology, developing a biological profile comprising sex, age, stature, and population affinity is essential in cases involving unidentified human remains [1, 2]. Age estimation plays a critical role not only in human identification but also in the investigation of pathologies and characteristics associated with specific age groups, thereby offering information relevant to anthropology and related fields such as epidemiology [3].

It is well established that age estimation is considerably more reliable in young individuals than in adults, and the literature contains significantly more methods for non‐adults. Adult age estimation is complicated by factors including greater inter‐individual variation in degenerative changes compared to developmental ones, and the increased susceptibility of adult skeletal aging to genetic, environmental, and pathological influences [2, 4]. Furthermore, different anatomical regions reflect age‐related changes at varying rates depending on sex and other biological factors. Traditional methods usually focus on limited anatomical structures and lack a framework for combining multiple skeletal features into a multifactorial analysis capable of yielding accurate estimates [4].

To address these issues, Navega et al. [1] developed the DRNNAGE (Deep Random Neural Networks for Adult Skeletal Age‐at‐Death Estimation) software as a multifactorial tool for adult age estimation. This system treats the entire skeleton as a single complex biomarker. By simultaneously analyzing dozens of traits, the neural network learns the non‐linear patterns of skeletal senescence across the lifespan. The DRNNAGE method relies on a machine learning model known as a Deep Random Neural Network (DRNN). Unlike conventional neural networks requiring computationally expensive training, the DRNN model utilizes a highly efficient regression‐based approach where only the final output layer is trained on known data. This model applies a macroscopic scoring system including up to 64 skeletal traits from the axial and appendicular skeleton, incorporating traditional and under‐explored age indicators.

The software is provided as an intuitive online Graphical User Interface (GUI) and can also be deployed offline as an R package. The project is fully open source, with both underlying code and anonymized reference data publicly available. A key advantage is its flexibility, as it can generate age estimates from incomplete sets of traits, making it practical for real‐world forensic casework where preservation is often poor. Additionally, the core functions exist as independent modular components, allowing researchers to programmatically replicate and automate the analysis logic for processing multiple samples.

The tool demonstrated precision and accuracy in the population for which it was developed [1]. However, a major limitation of age‐estimation methods is the substantial variation in aging processes across populations. Consequently, most tools are population‐specific and may not generalize well to other groups. Studies indicate that applying a method to a population different from its development sample can produce inconsistent relationships between chronological and skeletal age [2, 5, 6]. As stated by Cunha et al. [7], the best method is not necessarily the one with the highest accuracy, but the one validated across varied scenarios, large samples, and different populations while remaining practical and accessible. This highlights the need for validating DRNNAGE in diverse populations [7, 8], as deep neural network models represent an increasingly promising tool in forensic sciences [1, 9, 10].

Given these considerations, the objective of this study was to evaluate the performance of DRNNAGE in estimating age‐at‐death in an adult sample from Northeastern Brazil, aiming to assess its applicability within the country's forensic anthropology context.

## 2 MATERIALS AND METHODS

This research complied with national and institutional ethical standards for studies involving human subjects and received approval from the Research Ethics Committee. It is an analytical, quantitative, cross‐sectional study conducted at the Center for Studies in Forensic Anthropology (CEAF/UPE), in Recife, Pernambuco, Brazil.

The study population comprised identified human skeletons from the CEAF/UPE osteological collection, totaling 471 skeletons (ages 0–109 years), buried between 2011 and 2016 and exhumed between 2013 and 2019 [11]. Of this total, 244 skeletons belonged to males and 227 to females. For this study, 412 adult skeletons (≥20 years) were initially included and examined macroscopically according to the criteria outlined by Navega, Costa, and Cunha [1].

Skeletons showing trauma, taphonomic alterations, anomalies, or pathologies that compromised bone surfaces were excluded. Skeletons with insufficient analyzable markers (fewer than six of the 64 DRNNAGE markers) were also excluded. The final sample consisted of 400 skeletons (200 females and 200 males).

The software evaluates morphological traits of cranial sutures (palatine, coronal, sagittal, and lambdoid), vertebrae (cervical, lumbar, and S1), upper and lower limbs, fusion of the first and second sacral vertebrae, clavicles, first rib, pubic symphysis, sacroiliac joint, and acetabulum. Detailed trait descriptions and the scoring system are available within the software.

A preliminary training phase was conducted to assess scoring consistency. Random skeletons were examined by the primary researcher and then compared with assessments from a second examiner experienced in forensic anthropology. Cohen's Kappa was applied to test inter‐ and intra‐observer agreement according to Landis and Koch [12].

All bones were examined macroscopically in anatomical position, and data were recorded in spreadsheets. Analyses were performed using IBM™ SPSS® (version 22.0), R (version 4.3.3), and RStudio (version 2023.12.1).

Descriptive statistics included frequencies for categorical variables and means, medians, and standard deviations for age. Cohen's Kappa assessed observer agreement. Inferential analyses included Cramer's V to test the relationship between sex and morphological traits, and a modified Kolmogorov–Smirnov test to evaluate sex effects on age distributions. Antimeric asymmetry was tested using a chi‐square‐based procedure, and its effect on age distribution was evaluated using a modified Kolmogorov–Smirnov test. Left‐side traits were prioritized unless missing, in which case right‐side scores were used. No imputation procedures (statistical reconstruction of missing values) were performed.

Age estimation was performed using DRNNAGE (version 0.0.1.0) with default neural network parameters. The default parameters were predefined to provide the user with minimal technical overhead and represent a configuration extensively analyzed by the developer. The parameters used were: Network Algorithm equal to “Ensembled Randomized Network,” Layer Size equal to 32, Network Depth equal to 8, Gaussian Noise equal to 1, Uncertainty Level (alpha) equal to 0.1, Variance Model Exponent equal to 1, and RNG Seed equal to 99,676. Age estimates were obtained using consensus‐based deep random neural networks, with 8 layers of depth composed of 32 neurons (ReLU, rectifier linear units), with Gaussian regularization and a linear regime in the inference of predictive intervals for a truncated Gaussian model and a conformal prediction regressor. The pseudo‐random parameters of the system were controlled with seed 99,676 (Mersenne‐Twister generator).

To rigorously assess the performance of the age estimation model, we adopted the statistical framework proposed by Navega et al. [1]. This framework evaluates the model against four key parameters: accuracy, bias, validity, and efficiency.

Accuracy refers to the model's ability to predict an individual's age with minimal error. It was quantified using the Mean Absolute Error (MAE), which represents the average absolute difference between the known age and the estimated age.

Bias refers to the presence of systematic error in the model's predictions. In age estimation, a common pattern of bias is the overestimation of age for young individuals and the underestimation of age for old individuals. To measure this, we calculated the bias score (*β*e), defined as the slope of the regression line between the prediction errors and the known chronological ages. A score close to zero indicates a lack of systematic bias, while a positive slope indicates the typical age‐mimicry bias. For age‐stratified analyses, bias is also reported as the mean of the signed errors (i.e., the mean error).

Validity assesses whether the model's prediction intervals are reliable. This is measured by coverage, which is the percentage of individuals in the sample for whom the true chronological age falls within the calculated prediction interval. For a model to be considered valid, its calculated coverage should not depart abruptly from the nominal confidence level of the interval (e.g., a 95% prediction interval should ideally contain the true age for approximately 95% of the cases).

Efficiency relates to the tightness of the prediction intervals. A model is considered more efficient if it produces narrower prediction intervals while maintaining high validity. Efficiency was measured by analyzing the Predictive Interval Width (PIW), reported as the median width (difference between the interval endpoints) to assess the model's overall precision.

For age‐stratified analysis in addition to the metrics mentioned to assess performance, it also computed the average of point estimate, lower and upper bounds of the prediction interval obtained for the individuals in a given age interval. This allows assessment of the alignment of the obtained age estimate for a particular age group.

In this study, we approach DRNNAGE as a multifactorial tool and have limited our performance assessment to this specific scenario. Although DRNNAGE is capable of producing age estimates from single anatomical structures (e.g., the pubic symphysis alone), this functionality was not evaluated in the present study. That is, something is possible due the flexibility of the tool, not its underlying philosophy which advocate the usage of multiple and diverse skeletal traits.

Our analysis focused on the performance of three distinct multifactorial models:

1. A comprehensive model utilizing all available skeletal traits (up to *m* = 64)
2. A model based on a collection of classical age indicators (i.e., including cranial sutures, the pubic symphysis, the acetabulum, etc.).
3. A model based exclusively on degenerative traits of the axial and appendicular skeleton.

We conducted a global and age‐stratified (10‐year interval) analysis of the performance of this tool both for sex‐pooled and sex‐stratified samples.

## 3 RESULTS

The final sample comprised 400 identified adult skeletons (200 females and 200 males) with a mean age of 57.35 years (SD = 18.32; range = 20–99 years). The female subsample had a mean age of 56.88 years (SD = 18.41; range = 20–96 years), while the male subsample had a mean age of 57.82 years (SD = 18.27; range = 20–99 years). No significant difference in age distribution was observed between sexes (*p* = 0.682).

Inter‐observer agreement ranged from 0.50 to 1.00 (mean = 0.797; SD = 0.158), and intra‐observer agreement ranged from 0.66 to 1.00 (mean = 0.900; SD = 0.095). These values indicate substantial to excellent agreement, demonstrating high reproducibility and reliability of trait scoring.

Cramer's V analysis revealed no significant relationship between sex and morphological trait classification (V = 0.089; *p* = 0.078). Similarly, no significant difference was observed in age distribution between sexes (modified Kolmogorov–Smirnov test; *p* = 0.682). Antimeric analysis showed no significant asymmetry between left and right sides (chi‐square–based procedure; *p* = 0.156), and no significant effect of antimeric variation on age distribution (modified Kolmogorov–Smirnov test; *p* = 0.412).

**FIGURE 1:**
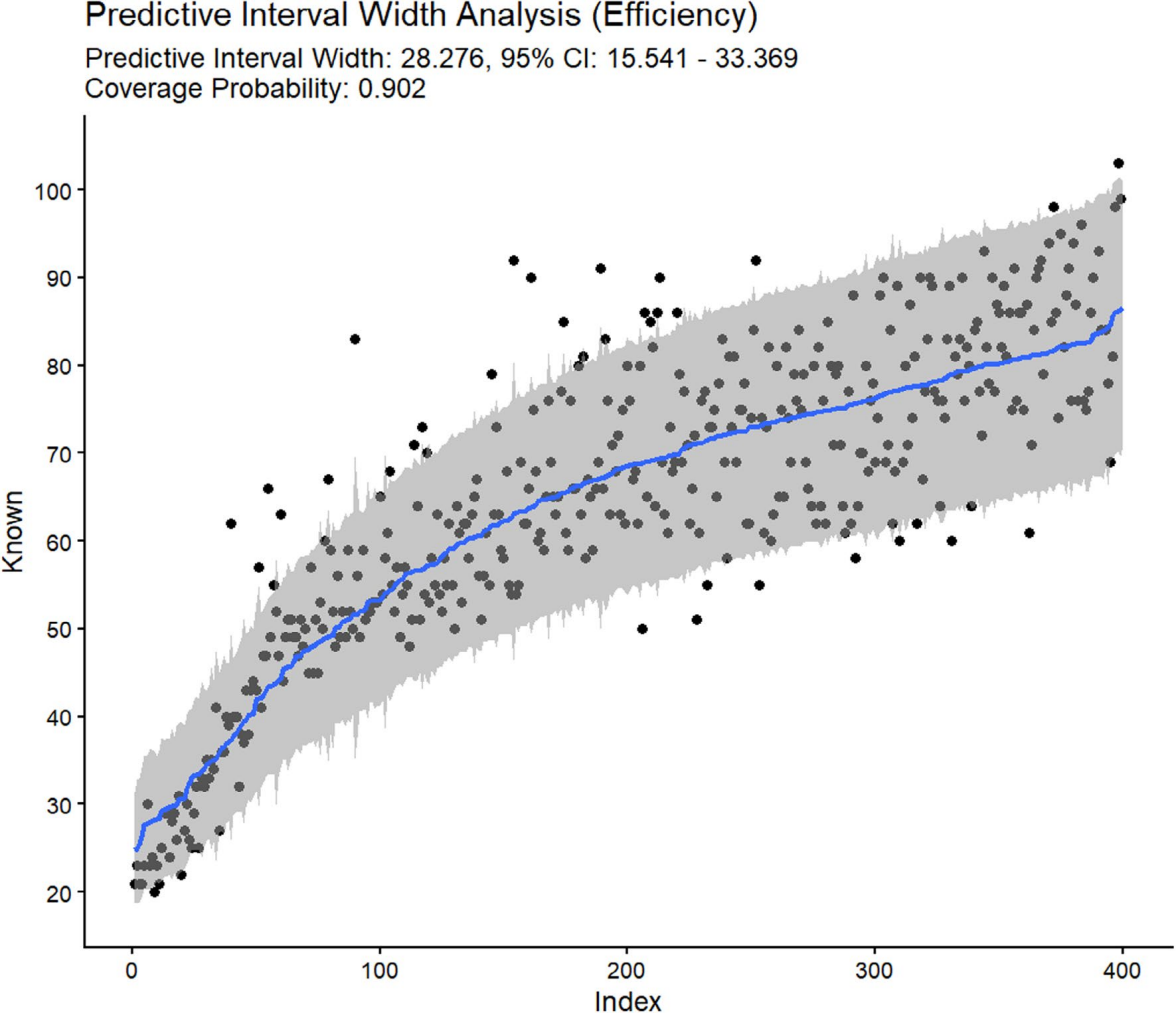
Analysis of predictive efficiency, displaying the distribution of predictive interval widths (Median PIW: 28.276; 95% Empirical Range: 15.541–33.369) and the overall empirical coverage (0.902) for nominal 90% prediction intervals (*n* = 400).

**FIGURE 2:**
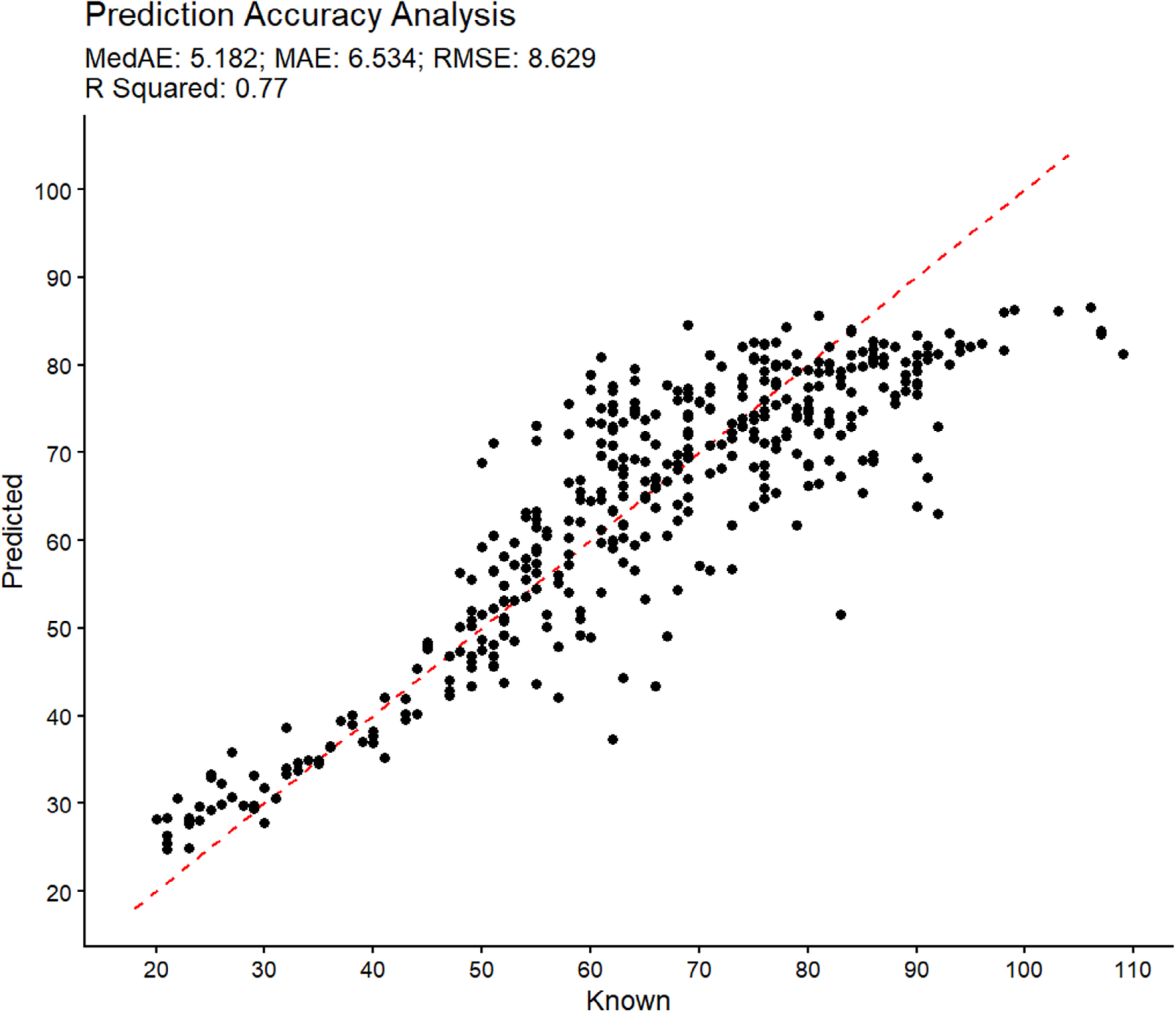
Prediction accuracy analysis illustrating the correlation between known chronological age and the age estimated by DRNNAGE.

**FIGURE 3:**
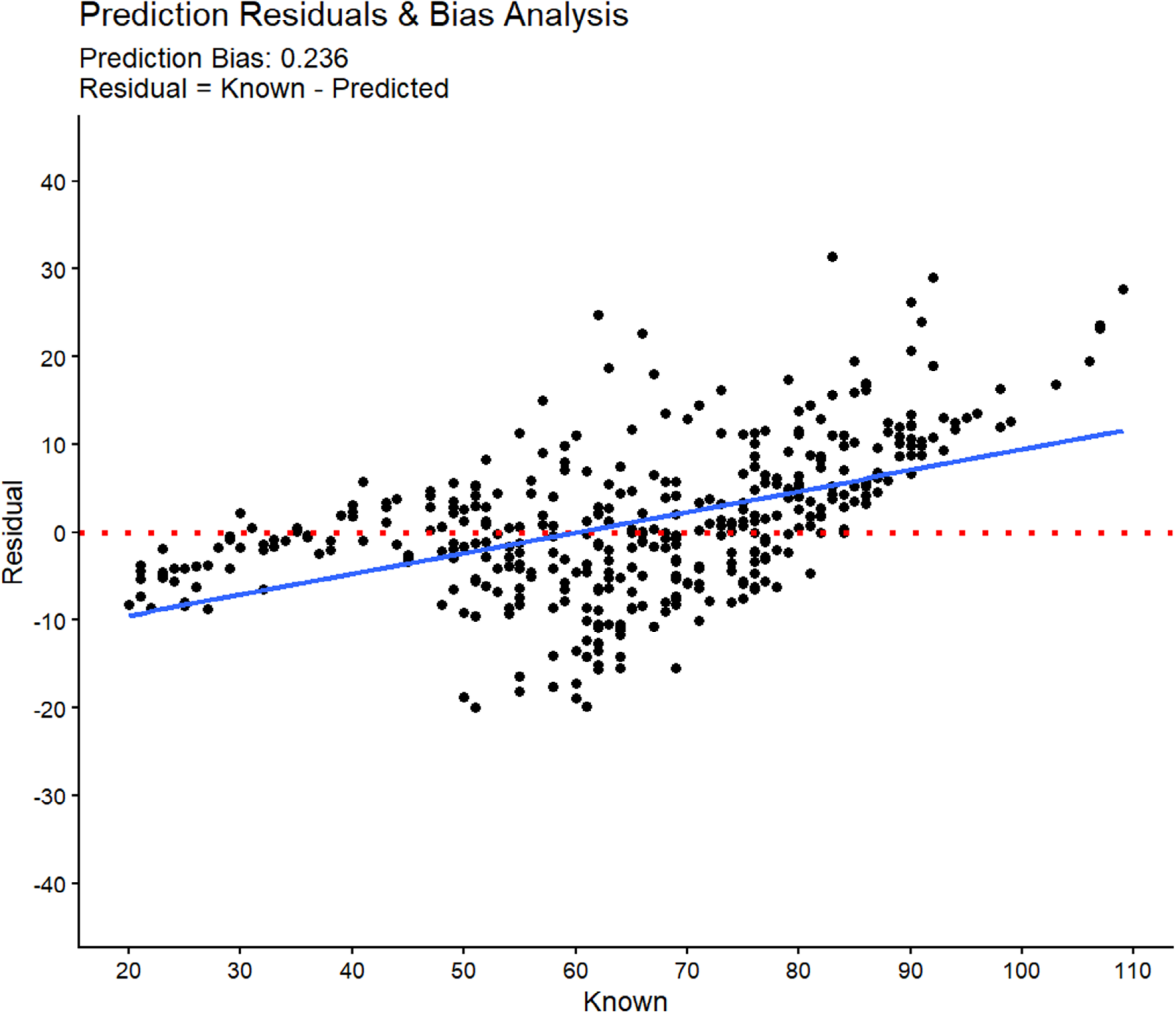
Analysis of prediction residuals and systematic bias trends across the lifespan.

The performance of the DRNNAGE method can be first evaluated visually to assess validity, accuracy, and bias patterns across the total sample (*n* = 400). Figure 1 illustrates the predictive interval width (efficiency) and coverage probability. The method demonstrated a high degree of validity, with 90.2% of individuals falling within the calculated prediction intervals. Figure 2 displays the relationship between known and predicted ages, revealing a strong correlation (*R* ^2^ = 0.77) and a Mean Absolute Error (MAE) of 6.534 years. The distribution of residuals is depicted in Figure 3, which highlights a systematic bias of 0.236 years overall. As seen in the residuals plot, the model exhibits the typical age estimation pattern: a tendency to overestimate younger individuals and underestimate older individuals, with the magnitude of error increasing at the extremes of the lifespan.

Detailed performance metrics for the full sample and sex‐specific subsets are summarized in Table 1. When utilizing all available traits (up to *m* = 64), the method achieved a grouped MAE of 6.54 years and a median Predictive Interval Width (PIW) of 28.28 years. Table 1 also highlights the model's heteroscedasticity the varying levels of uncertainty across the population. For the full trait set, the PIW ranged from 15.54 years to 33.37 (2.5% and 97.5% empirical quantiles), indicating that while the model generates precise estimates for robust cases, it appropriately widens the intervals for more ambiguous profiles.

**TABLE 1:** Accuracy, mean absolute error, coefficient of determination, and bias of DRNNAGE for the total sample.

	*n*	Available traits		MAE	Bias (*β*e)	Coverage (%)	Predictive interval width		
		*Q* (0.5) (%)	*Q* (0.025) (%)				*Q* (0.5)	*Q* (0.025)	*Q* (0.975)
All DRNNAGE traits (m = 64)									
Grouped	400	82.8	34.4	6.54	0.236	90.2	28.28	15.54	33.37
Female	200	78.12	31.2	8.09	0.308	85.5	29.36	18.12	34.03
Male	200	85.9	42.2	5.86	0.056	94.0	26.58	15.11	31.53
Classic traits only (*m* = 25)									
Grouped	400	87.5	33.3	7.16	0.242	92.2	34.33	14.60	45.208
Female	200	87.5	33.13	8.08	0.322	90.5	35.89	18.05	55.15
Male	200	91.7	45.7	6.23	0.104	94.0	31.98	13.32	43.16
Degenerative traits only (*m* = 39)									
Grouped	400	80.0	30.0	7.41	0.243	88.0	28.78	24.19	33.55
Female	200	75.0	24.93	8.60	0.358	90.5	28.98	24.60	34.22
Male	200	82.5	39.39	6.23	0.130	94.0	28.46	24.13	31.35

*Note*: *Q* (0.5), *Q* (0.025), *Q* (0.975) represent empirical quantiles.

Robustness regarding data completeness was also evident in Table 1. The median trait availability for the grouped sample was 82.8%. Notably, the lower bound of availability was 34.4%, demonstrating the software's capacity to generate estimates even when approximately two‐thirds of traits are missing. Sex‐stratified analysis in Table 1 revealed that males (*n* = 200) consistently outperformed females (*n* = 200). Males achieved an MAE of 5.86 years and 94.0% coverage, compared to females with an MAE of 8.09 years and 85.5% coverage. This disparity corresponds with preservation differences; while median availability was similar, the lower bound for males (42.2%) was higher than for females (31.2%), suggesting female skeletons in the lower quartiles were more fragmentary.

The performance of the model across specific age cohorts for the grouped sample is detailed in Table 2. In the youngest cohort (20–30 years), the model demonstrated high precision with a median PIW of 15.83 years and 91.7% coverage. Reliability remained peak in the 30–40 and 40–50 intervals, both achieving 100% coverage. However, performance declined in advanced age groups. The 90–100 cohort showed a significant negative bias of −13.85 years, and the model failed to correctly classify any individuals in the 100–110 range (0.0% coverage), reflecting the ceiling effect of skeletal markers.

**TABLE 2:** Information regarding age estimation separated by age intervals, regardless of sex.

Age interval	*n*	Traits (%)	MAE	Bias (*β*e)	Coverage (%)	Predictive interval width (median)	Point estimate	Lower (90% PI)	Upper (90% PI)
[20,30)	24	88.7	4.79	4.79	91.7	15.83	29.41	21.72	37.54
[30,40)	17	75.8	1.58	0.97	100.0	18.63	35.15	25.92	44.55
[40,50)	28	82.4	3.05	−0.59	100.0	21.14	45.05	34.56	55.71
[50,60)	68	75.0	5.69	2.22	89.7	25.11	56.64	44.17	69.28
[60,70]	96	80.3	7.12	3.08	89.6	27.75	67.61	53.79	81.54
[70,80)	74	71.7	4.97	−1.10	95.9	29.89	73.89	58.95	88.85
[80,90)	63	75.1	7.79	−7.64	90.5	30.63	76.11	60.81	91.44
[90,100)	25	72.3	13.85	−13.85	76.0	31.25	78.55	62.83	94.07
[100,110]	5	60.9	22.12	−22.12	0.0	31.43	84.28	68.14	99.57

Sex‐specific age patterns are presented in Tables 3 and 4. Females, detailed in Table 3, showed lower reliability in the youngest cohort (20–30 years) with 66.7% coverage and a systematic bias of 7.27 years. In contrast, males, shown in Table 4, achieved 100% coverage and a lower bias (5.60 years) in the same 20–30 interval. Conversely, males exhibited a steeper decline in the 90–100 age group (66.7% coverage) compared to females (77.3% coverage) in the same bracket.

**TABLE 3:** Information regarding age estimation of female individuals divided by age intervals.

Sex	Age interval	*n*	Traits (%)	MAE	Bias (*β*e)	Coverage (%)	Predictive interval width (median)	Point estimate	Lower (90% PI)	Upper (90% PI)
Female	[20,30)	3	69.8	7.27	7.27	66.7	15.89	30.60	22.74	38.62
Female	[30,40)	5	75.6	1.66	0.02	100.0	18.96	34.62	25.22	44.18
Female	[40,50)	9	87.3	2.75	0.34	100.0	21.33	46.56	35.96	57.29
Female	[50,60)	29	72.5	6.14	0.91	86.2	25.05	55.26	42.83	67.88
Female	[60,70]	41	77.4	6.87	2.80	92.7	28.00	67.46	53.53	81.53
Female	[70,80)	41	68.0	5.34	−1.58	95.1	29.88	73.37	58.41	88.29
Female	[80,90)	45	73.5	8.45	−8.24	88.9	30.69	75.45	60.13	90.82
Female	[90,100)	22	70.0	13.89	−13.89	77.3	31.38	78.33	62.57	93.95
Female	[100,110]	5	60.9	22.12	−22.12	0.0	31.43	84.28	68.14	99.57

**TABLE 4:** Information regarding age estimation of male individuals divided by age intervals.

Sex	Age interval	*n*	Traits (%)	MAE	Bias (*β*e)	Coverage (%)	Predictive interval width (median)	Point estimate	Lower (90% PI)	Upper (90% PI)
Male	[20,30)	21	91.7	5.60	5.60	100.0	24.32	30.41	19.34	43.66
Male	[30,40)	12	74.7	1.58	−0.72	100.0	25.13	33.28	21.56	46.68
Male	[40,50)	19	82.0	3.23	0.02	100.0	26.99	45.39	32.16	59.15
Male	[50,60)	39	82.2	6.95	4.09	84.6	28.31	58.58	44.69	73.00
Male	[60,70]	55	85.2	8.07	3.82	80.0	28.79	68.24	54.03	82.83
Male	[70,80)	33	80.1	5.79	−0.83	97.0	29.61	74.20	59.54	89.15
Male	[80,90)	18	84.3	5.59	−5.59	100.0	29.65	78.30	63.52	93.16
Male	[90,100)	3	95.8	13.75	−13.75	66.7	28.41	79.91	65.65	94.06

The utility of specific trait categories was evaluated by restricting the analysis to “classic” and “degenerative” markers. Table 5 presents the grouped results for classic traits (*m* = 25). While this subset maintained high overall coverage (92.2%), it was characterized by high heteroscedasticity, with PIW ranging from 14.60 years to 45.21 years (2.5% and 97.5% empirical quantiles), indicating a significant loss of precision for difficult cases compared to the full model.

**TABLE 5:** Information regarding age estimates separated by age intervals, regardless of sex, considering only classic traits (*m* = 25).

Age interval	*n*	Traits (%)	MAE	Bias (*β*e)	Coverage (%)	Predictive interval width (median)	Point estimate	Lower (90% PI)	Upper (90% PI)
[20,30)	24	89.8	4.99	4.99	70.8	15.56	29.62	22.06	37.63
[30,40)	17	76.2	3.27	1.71	100.0	19.66	35.89	26.19	45.85
[40,50)	28	84.7	4.71	−2.07	96.4	22.54	43.58	32.51	55.05
[50,60)	68	79.5	7.29	3.24	95.6	30.71	57.66	42.45	73.16
[60,70]	96	82.7	7.72	2.71	91.7	33.69	67.23	50.41	84.10
[70,80)	74	76.1	4.78	0.48	100.0	37.29	75.47	56.58	93.87
[80,90)	63	80.1	6.96	−6.79	98.4	37.84	76.96	57.83	95.67
[90,100)	25	75.4	15.68	−15.68	76.0	38.52	76.72	57.21	95.73
[100,110]	5	59.2	27.14	−27.14	0.0	38.65	79.26	59.30	97.95

Grouped results for degenerative traits (*m* = 39) are shown in Table 6. These traits proved highly effective for younger individuals, achieving 100% coverage across all intervals between 20 and 50 years. However, this high coverage comes at the cost of specificity, as reflected in the predictive interval widths. For the 20–30 age group, the median PIW was 24.27 years, with a 90% prediction interval ranging from 19.35 to 43.62 years. Similarly, in the 40–50 group, while coverage remained perfect, the upper bound of the interval extended to 60.11 years (Median PIW: 27.06 years), indicating a wider range of uncertainty.

**TABLE 6:** Information regarding age estimation divided by age group intervals, regardless of sex, considering only degenerative traits (*m* = 39).

Age interval	*n*	Traits (%)	MAE	Bias (*β*e)	Coverage (%)	Width	Point estimate	Lower (90% PI)	Upper (90% PI)
[20,30)	24	90.1	5.76	5.76	100.0	24.27	30.38	19.35	43.62
[30,40)	17	76.7	2.11	−0.32	100.0	25.34	33.86	22.02	47.36
[40,50)	28	85.0	3.28	0.68	100.0	27.06	46.32	33.05	60.11
[50,60)	68	80.1	7.98	3.66	83.8	28.48	58.09	44.17	72.65
[60,70]	96	83.0	8.49	4.32	79.2	28.97	68.84	54.60	83.58
[70,80)	74	76.8	5.78	−0.95	97.3	29.79	74.03	59.32	89.11
[80,90)	63	80.6	7.46	−7.04	93.7	29.96	76.71	61.87	91.83
[90,100)	25	75.7	13.54	−13.54	76.0	30.32	78.86	63.76	94.08
[100,110]	5	59.2	21.22	−21.22	0.0	29.66	85.18	70.13	99.79

Furthermore, their utility diminished sharply in senescence, with coverage dropping to 0.0% for the 100–110 age group. This lack of coverage is primarily due to the model's incapacity to capture the upper part of the predictive interval; the upper boundary reached only 99.79 years, falling short of the actual age of the individuals in this cohort. Despite this interval ceiling, the point estimate (85.18 years) and the lower boundary of the interval (70.13 years) were clearly aligned with the advanced age of the sample, indicating that the model correctly identified the senescence of these individuals even if the specific interval failed to encompass the extreme chronological age.

Sex‐specific analyses of these subsets are detailed in Tables 7, 8, 9 through 10. For females using classic traits (Table 7), the model achieved 100% coverage in the 30–50 and 70–80 intervals. For males using classic traits (Table 8), precision was notably penalized in the oldest cohort; the median PIW for the 90–100 group was 36.70 years, significantly wider than the 28.41 years achieved with the full model. Regarding degenerative traits, females (Table 9) maintained 100% coverage from 20 to 50 years. Similarly, males (Table 10) achieved 100% coverage in the 20–50 range, as well as in the 80–90 interval, suggesting that degenerative markers retain specific utility for aging males that was not observed in the female subsample.

**TABLE 7:** Information regarding age estimation of female individuals divided by age intervals, considering only classic traits (*m* = 25).

Sex	Age interval	*n*	Traits (%)	MAE	Bias (*β*e)	Coverage (%)	Width	Point estimate	Lower (90% PI)	Upper (90% PI)
Female	[20,30)	3	77.3	6.83	6.83	33.3	16.23	30.17	22.26	38.49
Female	[30,40)	5	80.8	4.41	−0.56	100.0	19.44	34.04	24.57	44.01
Female	[40,50)	9	91.1	5.26	−2.34	100.0	22.78	43.88	32.62	55.40
Female	[50,60)	29	76.7	7.18	1.37	96.6	30.77	55.71	40.53	71.30
Female	[60,70]	41	79.7	7.19	0.43	90.2	33.95	65.09	48.14	82.09
Female	[70,80)	41	73.3	5.17	−0.77	100.0	37.43	74.18	55.19	92.62
Female	[80,90)	45	78.6	7.30	−7.20	97.8	38.17	76.49	57.21	95.38
Female	[90,100)	22	72.5	15.81	−15.81	72.7	38.77	76.42	56.78	95.55
Female	[100,110]	5	59.2	27.14	−27.14	0.0	38.65	79.26	59.30	97.95

**TABLE 8:** Information regarding age estimation of male individuals divided by age intervals, considering only classic traits (*m* = 25).

Sex	Age interval	*n*	Traits (%)	MAE	Bias (*β*e)	Coverage (%)	Width	Point estimate	Lower (90% PI)	Upper (90% PI)
Male	[20,30)	21	91.6	4.73	4.73	76.2	15.47	29.54	22.04	37.51
Male	[30,40)	12	74.3	2.80	2,66	100.0	19.74	36.66	26.87	46.62
Male	[40,50)	19	81.7	4.45	−1.93	100.0	22.43	44.43	32.45	54.88
Male	[50,60)	39	81.6	7.36	4.63	84.6	30.66	59.12	43.88	74.54
Male	[60,70]	55	84.9	8.11	4.40	80.0	33.50	68.82	52.09	85.59
Male	[70,80)	33	79.5	4.30	2.05	97.0	37.12	77.08	58.30	95.43
Male	[80,90)	18	84.0	6.11	−5.75	100.0	37.02	78.14	59.37	96.39
Male	[90,100)	3	96.0	14.78	−14.78	66.7	36.70	78.89	60.35	97.05

**TABLE 9:** Information regarding age estimation of female individuals divided by age intervals, considering only degenerative traits (*m* = 39).

Sex	Age interval	*n*	Traits (%)	MAE	Bias (*β*e)	Coverage (%)	Width	Point estimate	Lower (90% PI)	Upper (90% PI)
Female	[20,30)	3	79.2	6.88	6.88	100.0	23.88	30.21	19.42	43.30
Female	[30,40)	5	81.7	3.40	0.65	100.0	25.84	35.25	23.13	48.97
Female	[40,50)	9	91.2	3.39	2.09	100.0	27.22	48.28	34.92	62.14
Female	[50,60)	29	77.3	9.36	3.08	82.8	28.70	57.43	43.47	72.17
Female	[60,70]	41	80.1	9.04	4.99	78.0	29.21	69.65	55.37	84.58
Female	[70,80)	41	74.2	5.77	−1.06	97.6	29.94	73.89	59.15	89.08
Female	[80,90)	45	79.1	8.21	−7.62	91.1	30.09	76.07	61.21	91.30
Female	[90,100)	22	72.9	13.51	−13.51	77.3	30.58	78.72	63.50	94.09
Female	[100,110]	5	59.2	21.22	−21.22	0.0	29.66	85.18	70.13	99.70

**TABLE 10:** Information regarding age estimation of male individuals divided by age intervals, considering only degenerative traits (*m* = 39).

Sex	Age interval	*n*	Traits (%)	MAE	Bias (*β*e)	Coverage (%)	Width	Point estimate	Lower (90% PI)	Upper (90% PI)
Male	[20,30)	21	91.7	5.60	5.60	100.0	24.32	30.41	19.34	43.66
Male	[30,40)	12	74.7	1.58	−0,72	100.0	25.13	33.28	21.56	46.68
Male	[40,50)	19	82.0	3.23	0.02	100.0	26.99	45.39	32.16	59.15
Male	[50,60)	39	82.2	6.95	4.09	84.6	28.31	58.58	44.69	73.00
Male	[60,70]	55	85.2	8.07	3.82	80.0	28.79	68.24	54.03	82.83
Male	[70,80)	33	80.1	5.79	−0.83	97.0	29.61	74.20	59.54	89.15
Male	[80,90)	18	84.3	5.59	−5.59	100.0	29.65	78.30	63.52	93.16
Male	[90,100)	3	95.8	13.75	−13.75	66.7	28.41	79.91	65.65	94.06

## 4 DISCUSSION

The human identification process involving skeletal remains is inherently challenging and requires methods that are rigorously tested, accurate, and reproducible [13]. In this study, the analysis of skeletal markers demonstrated high intra‐observer agreement (mean = 0.900) and substantial inter‐observer agreement (mean = 0.797). These findings reflect excellent repeatability and reproducibility and align with those reported by the developers of DRNNAGE [1]. Rizos et al. [14] found lower agreement values in a Greek sample (0.748 for intra‐observer agreement and 0.615 for inter‐observer agreement), a difference that is expected considering population‐specific aging patterns [2, 5, 6, 15], which reinforces the relevance of the present study performed in a population of Northeast Brazil.

The overall coverage of 90.2% (aligned with nominal confidence level tested) and MAE of 6.54 years for the Brazilian sample indicate that DRNNAGE performs well in this population. Coverage differed between sexes, with higher performance for males (94.0%) than for females (85.5%). This difference can be attributable to preservation disparities. While median trait availability was comparable between sexes, the lower bound of availability was considerably higher for males (42.2%) than for females (31.2%). This indicates that poorly preserved female skeletons were more fragmentary/incomplete than their male counterparts, directly impacting the model's performance in those cases. These results are more promising than those found by Rizos et al. [14], who obtained a coverage of 46.1% for the total sample, 52% for females, and 41.3% for males, and less favorable when compared to those of Navega et al. [1], who found an average absolute error of 5.69. As mentioned before, it is not uncommon to find divergent results for different populations; however, considering that Rizos et al. [14] and Navega et al. [1] studied European samples, such a discrepancy between them may be different from what is expected [16].

Comparing these results presented by DRNNAGE in the Brazilian sample with other age estimation methods also based on the analysis of dry bone morphology, it is noted that the results are also promising. For example, Luna and Aranda [17], using the analysis of the first rib, achieved 43.51% of correct estimates for the total sample. Kotěrová et al. [2], in turn, in a study conducted with osteological collections from various sources, found, through multilinear regression analysis of markers of the pubic symphysis and sacroiliac joint, an absolute mean error of 9.7. Furthermore, it is worth noting that several collections analyzed in the study by Kotěrová et al. [2] include individuals who lived in the 19th century, which reinforces the importance of new research, such as ours, with a contemporary population, to obtain results that are more reliable for the current reality.

In 2009, Cunha et al. [7] recommended the use of methods based on the analysis of the pubic symphysis, the sternal end of the fourth rib, the auricular surface of the iliac bone, and degenerative dental characteristics to estimate the age of adult skeletons. Garvin and Passalacqua [18] found, through a questionnaire applied to 145 forensic anthropologists in which five anatomical traits were proposed, that for estimating the age of adult individuals, the most commonly used markers were the pubic symphysis, the sternal end of the rib, and the auricular surface of the ilium, which is consistent with the recommendations of the 2009 study. Bailey and Vidoli [19], in turn, found the auricular surface of the ilium to be the most promising marker, with an accuracy of 88%. These data reinforce our findings that DRNNAGE demonstrated comparable or superior performance relative to methods most frequently used by forensic anthropologists for estimating the age of adults.

When separating the sample by sex and age groups, results similar to those found by Navega, Costa, and Cunha [1] are observed, with accurate results for individuals under 50 years of age. The sole exception was female skeletons aged 20 to 30, which had a coverage of 66.7% and represent a total of three individuals in the sample. Additionally, male individuals between 80 and 90 years of age obtained a percentage of 100% in their estimates. As for individuals in the 100 to 110 age group, the software failed to correctly allocate any cases (0% coverage), producing a substantial bias of −22.12 years. This systematic underestimation confirms that the software's internal limit (104 years) acts as a ceiling, compromising the accuracy and reliability of DRNNAGE for centenarians.

Considering that in practical cases it is common for not all bones of the skeleton to be present, performing an estimate with fewer traits becomes interesting. Given this, when separating the analyses into classic age traits, it was observed that DRNNAGE continued to perform satisfactorily, with an accuracy of 92.2% and an MAE of 7.16 years. However, this subset exhibited high heteroscedasticity, with predictive interval widths ranging widely from 14.60 years to 45.21 years. This corroborates the hypothesis that estimates based on a single variable or on certain bones are reliable but sometimes provide a wide possible age range, especially in individuals after the 5th decade of life, which could be reduced by a multifactorial analysis of the skeleton [17, 20, 21].

When the analysis is performed only with age‐related degenerative traits, the model maintains high validity (achieving 100% coverage in ages 20–50) but exhibits reduced efficiency compared to the full model. This suggests that while degenerative traits are highly consistent in younger skeletons (homoscedasticity), they are less informative than classic traits for specific estimation, resulting in wider intervals. Furthermore, accuracy diminished sharply in the oldest cohorts, with coverage dropping to 0.0% for the 100–110 age group due to the model's incapacity to capture the upper limit of the predictive interval.

In addition, performance remained satisfactory even with significant data loss. The median trait availability for the sample was 82.8%, and the software successfully generated estimates even at the lower end of 34.4% availability. This robustness is particularly relevant for real forensic cases where skeletal completeness varies considerably.

Another relevant point to note is the underestimation of age in older individuals, especially those aged 80 and above. This decrease in the accuracy of age estimation in older individuals has also been reported in other studies [1, 7, 16], demonstrating that it is a common and current problem in age estimation in adults, which should be taken into account by forensic anthropologists. This downward trend is also observed in methods based on DNA methylation [22], demonstrating that estimating age in older adults remains a broad methodological challenge across multiple approaches.

In addition to studies based on bone traits, research analyzing dental information is also often of great value to the field of forensic anthropology. However, when comparing the present study with research conducted on the Brazilian adult population based on age‐related dental changes [23, 24], it is noted that the results obtained by DRNNAGE are more promising, presenting greater accuracy and consequently a lower mean absolute error.

Considering the practical forensic context in Brazil, DRNNAGE offers important advantages: it is freely accessible, does not require advanced imaging equipment, and can be applied directly to dry bone, making it highly suitable for routine casework in forensic institutes with limited resources. It is also pertinent to mention that the limitations encountered by the software must be observed and considered in practical cases, but they do not prevent its use and, as previously mentioned, are inherent to the difficulty of estimating age in adult individuals.

Overall, the software demonstrated a good performance in this Northeastern Brazilian sample. Given Brazil's continental dimensions and diverse population structure [25], further regional studies are recommended to assess nationwide applicability. Understanding intra‐ and inter‐population variability [16] remains essential for refining age‐estimation methods and ensuring their reliability across diverse forensic contexts. Finally, the stratified analysis of very advanced age suggests the need for a reframing of how results are reported for the oldest cohorts and its performance measured. The complete failure to capture the 100–110 age group (0.0% coverage) is driven primarily by the software's incapacity to extend the upper prediction limit beyond its training ceiling (104); for instance, the upper boundary for this group reached only 99.57 years using all traits (Table 2) and 99.79 years using degenerative traits (Table 6). Despite this interval failure, the model clearly signaled extreme senescence. The point estimates (84.28 years for the full model; 85.18 years for degenerative traits) and the lower bounds of the prediction intervals (68.14 years; 70.13 years) consistently situated these individuals in the highest biological age tier. Consequently, reporting for such cases should move beyond closed intervals. When a point estimate exceeds a high threshold, it may be more statistically robust to utilize a cumulative distribution function approach, reporting the age simply as greater than the lower prediction bound (e.g., “age >70 years”). This approach avoids the mechanical exclusion of centenarians caused by the interval ceiling while preserving the valid forensic intelligence provided by the lower limit. This is particularly relevant given the global increase in life expectancy and the increasing number of cases involving the elderly.

## FUNDING INFORMATION

David Senhora Navega is funded under Marie Skłodowska‐Curie Actions (MSCA), Grant Agreement ID 101209534, Funding Scheme HORIZON‐TMA‐MSCA‐PF‐EF—HORIZON TMA MSCA Postdoctoral Fellowships—European Fellowships.

## CONFLICT OF INTEREST STATEMENT

The authors have no conflicts of interest to declare.

## Data Availability

Data available upon reasonable request.
